# Sleep, inflammation, and hemodynamics in rodent models of traumatic brain injury

**DOI:** 10.3389/fnins.2024.1361014

**Published:** 2024-02-15

**Authors:** Tabitha R. F. Green, Sean D. Carey, Grant Mannino, John A. Craig, Rachel K. Rowe, Mark R. Zielinski

**Affiliations:** ^1^Department of Integrative Physiology, University of Colorado Boulder, Boulder, CO, United States; ^2^Veterans Affairs (VA) Boston Healthcare System, West Roxbury, MA, United States; ^3^Department of Psychiatry, Harvard Medical School, West Roxbury, MA, United States

**Keywords:** sleep, hemodynamics, cerebral blood flow, neuroinflammation, TBI

## Abstract

Traumatic brain injury (TBI) can induce dysregulation of sleep. Sleep disturbances include hypersomnia and hyposomnia, sleep fragmentation, difficulty falling asleep, and altered electroencephalograms. TBI results in inflammation and altered hemodynamics, such as changes in blood brain barrier permeability and cerebral blood flow. Both inflammation and altered hemodynamics, which are known sleep regulators, contribute to sleep impairments post-TBI. TBIs are heterogenous in cause and biomechanics, which leads to different molecular and symptomatic outcomes. Animal models of TBI have been developed to model the heterogeneity of TBIs observed in the clinic. This review discusses the intricate relationship between sleep, inflammation, and hemodynamics in pre-clinical rodent models of TBI.

## 1 TBI as a disease process

**Figure 1:**
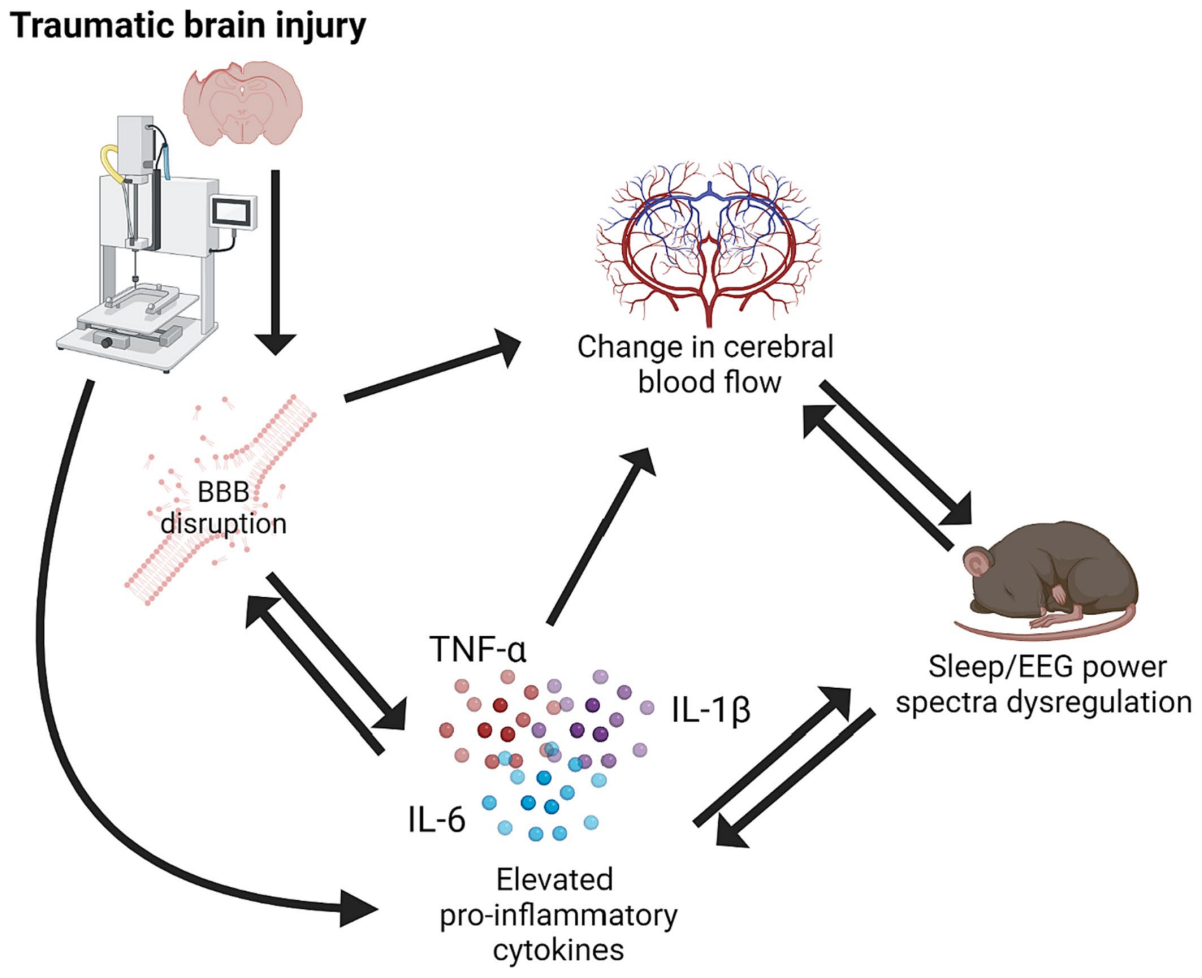
Schematic of relationship between traumatic brain injury, pro-inflammatory cytokines, BBB disruption, cerebral blood flow, and sleep/EEG power spectra dysregulation. Created using BioRender.com.

Traumatic brain injury (TBI) is a major cause of morbidity and mortality worldwide. TBI is caused by a mechanical insult to the brain that results in motor, cognitive, affective, and behavioral symptoms. Although TBI is a rapid onset condition, a complex disease process ensues and can progress chronically. TBI causes a robust inflammatory response triggered by tissue damage. Initially, inflammation can be beneficial and allow restoration and clean-up of damaged tissues. However, inflammation often progresses chronically post-TBI and creates a unique and progressive disease environment causing symptoms to evolve over time. Sleep is a biological system that is tightly coupled with inflammation due to the crossover in signaling molecules that are involved in both inflammatory and sleep processes. Furthermore, brain hemodynamics alter with sleep–wake state, which can become dysregulated post-TBI (Figure 1; Townsend et al., 1973; Madsen et al., 1991a,b; Bouma et al., 1992; Braun et al., 1997; Soustiel et al., 2005; Bangash et al., 2008; Salehi et al., 2017).

TBI can be broadly classified into three categories based on injury modality: diffuse, focal, and blast. These injury modalities are not mutually exclusive. Diffuse TBI is caused by the head or neck forcefully colliding with an object or surface. Common causes of diffuse TBI are falls, domestic violence, contact/collision sports, and motor vehicle accidents. In contrast, focal TBI results from a penetrating head wound, and common causes include gunshot wounds, skull fractures, and shrapnel injuries. Blast TBI results from shock waves, often associated with combat related explosions (Bass et al., 2012). Shock waves are transmitted through the brain causing direct movement and disruption of brain tissue (Säljö et al., 2008; Kovacs et al., 2014). Furthermore, shock waves cause displacement and oscillations of blood and other fluids in vessels throughout the body, which is a second, but simultaneous, cause of blast-induced TBI (Long et al., 2009; Kovacs et al., 2014).

A hallmark symptom of diffuse TBI is diffuse axonal injury. Diffuse axonal injury, characterized by damage to white matter tracts and axons, is present in all severities of diffuse TBI (Johnson et al., 2013). There are many pathological features of diffuse axonal injury that contribute to the acute and chronic symptoms associated with the condition, for example structural axonal damage, disrupted axonal transport, and axonal swelling (Johnson et al., 2013). Diffuse axonal injury can progress to Wallerian degeneration, a type of neurodegeneration associated with physical damage to the neuron (Johnson et al., 2013; Koliatsos and Alexandris, 2019). Diffuse TBI does not exhibit immediate cell death; however, neuronal cell death does occur over time because of secondary inflammatory processes (Lifshitz et al., 2007, 2016). In contrast, focal TBI is characterized by overt tissue disruption and cell death at the site of injury as early as 4–6 h post-TBI (Flygt et al., 2016; Díaz et al., 2023).

## 2 Pre-clinical models of TBI

**Figure 2:**
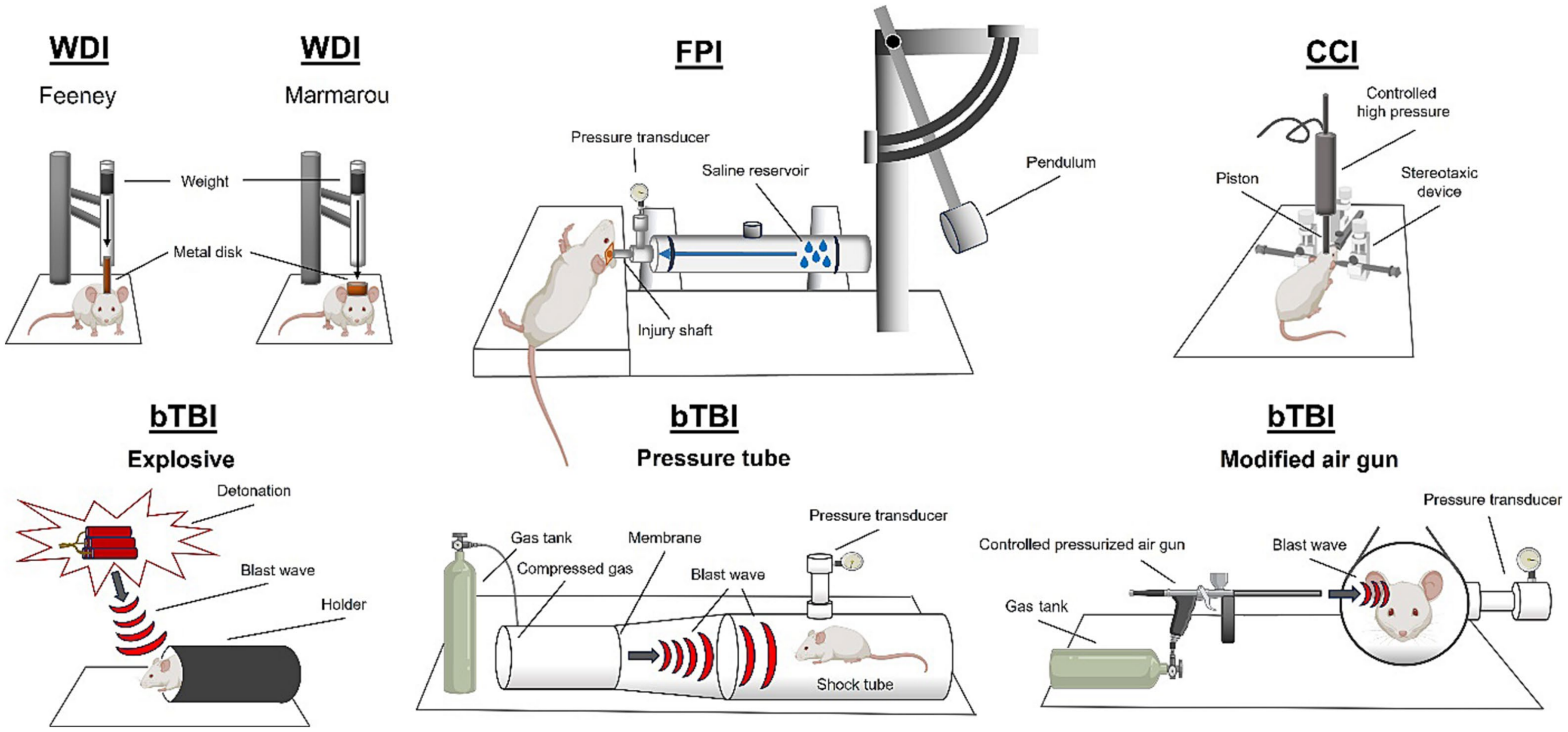
Rodent models of traumatic brain injury. Weight drop injury (WDI), fluid percussion injury (FPI), controlled cortical impact (CCI), and blast traumatic brain injury (bTBI). Created using BioRender.com.

TBI is often studied preclinically using animal models (Figure 2). Most commonly, rodent models are used, however, there are many other established models including ferrets, pigs, drosophila, cats, and rabbits (Ma et al., 2019). Diffuse TBI can be modeled using a closed head injury technique. Closed head TBI is often modeled using a weight drop technique. The weight drop injury (WDI) TBI model involves a gravity falling weight onto the head of a rodent where weight position and displacement of the force can be altered. Variations in WDI models include the Feeney model that causes a direct impact to the dura or the Marmarou model that induces a diffuse impact due to a plate placed above the skull (Feeney et al., 1981; Foda and Marmarou, 1994). The size of the object and the speed and height from which it is dropped change the severity and biomechanics of the weight drop generated injury (Bodnar et al., 2019). A controlled cortical impact (CCI) device can be used to deliver an impact to the intact skull causing acceleration/deceleration of the brain. Closed head piston driven methods can be performed with or without a rodent helmet and on either an intact scalp or exposed skull. For an in-depth review of closed head injury models refer to Bodnar et al. (2019). Another method to induce a diffuse TBI is midline fluid percussion injury (mFPI). To induce an mFPI, a craniectomy is performed on the sagittal suture between bregma and lambda markings on the skull. The fluid impulse is delivered to the intact dura through an injury hub assembly attached to the skull (Lifshitz et al., 2016). The forces applied to the brain are dissipated through the tissue and cause pathology at multiple sites, determined by the structural properties of the skull (Beitchman et al., 2021).

Focal TBI is most studied using the CCI method. CCI was designed to administer focal TBIs to Ferrets in 1988 (Lighthall, 1988), but since has been adapted to many species (primate, mouse, swine; Kobeissy, 2015). Most commonly, CCI is used in mice and rats and involves craniectomy surgery to remove a small portion of the parietal bone. After removal of the skull, a pneumatic (or electromagnetic) piston is driven into the brain tissue at the desired depth and velocity causing tissue disruption. Lateral fluid percussion injury (LFPI) results in focal brain damage at the site of the impact, and also causes diffuse pathology throughout the brain. LFPI requires a craniectomy in the parietal bone, and the assembly of an injury hub onto the skull. Using the fluid percussion device, a fluid impulse is delivered directly to the brain through the injury hub, onto the intact dura, which causes a focal lesion to the brain, as well as applying rotational forces to the brain that results in widespread diffuse pathology (Alder et al., 2011). The craniectomy site can be adjusted to change the location of the focal lesion (i.e., frontal bone; Ouyang et al., 2018).

To induce pathology associated with a blast, models of blast TBI (bTBI) use peak overpressure, and number of exposures as metrics (Skotak et al., 2019). Explosive bTBI methods can be modeled in rodents using an open field explosive device detonation which results in both primary and secondary blast pathology (Kovacs et al., 2014). To study the primary blast mechanism in the absence of secondary shockwave reverberations in the rat, blast tubes have been developed (Säljö et al., 2000; Risling et al., 2011). Blast tubes have also been invented to study blast injury in swine (Bauman et al., 2009). bTBI can also be induced in rodents using a pressure tube, in which build-up of compressed gas is used to rupture a breakable membrane which causes a shockwave to travel down the tube (Long et al., 2009; Reneer et al., 2011). Blast injury with secondary shrapnel penetration can be modeled using a blast gun (Gamboa et al., 2021). Additionally, blast injury can be modeled using a modified air gun to administer focal injury via directed pressurized air (Heldt et al., 2014).

## 3 Sleep dysregulation after TBI

Sleep disturbances occur in 30–84% of patients post-TBI (Paredes et al., 2021). TBIs can induce profound sleep dysregulation, including hypersomnia and hyposomnia, sleep fragmentation, sleepiness, difficulty falling asleep, and altered electroencephalograms (EEGs; Sandsmark et al., 2017; Aoun et al., 2019; Green et al., 2020; Rowe and Griesbach, 2022). In the clinic, patients report both acute and chronic sleep disturbances, and these occur following mild, moderate, and severe TBI (Verma et al., 2007; Viola-Saltzman and Watson, 2012; Wickwire et al., 2016; Mollayeva et al., 2017). Rodent models of TBI are used to investigate sleep disturbances. Experimental TBI results in acute and chronic sleep disturbances, and these sleep disturbances occur following both mild and moderate TBI (Table 1). It is important to note that there are multiple factors that can influence sleep disturbances including the location of the injury, severity, depth of impact, diffuse or focal, pressure of blast, and brain area of injury.

**Table 1:** Summary of key sleep and EEG findings of studies involving rodent TBI models.

Citation	TBI model	Animal	Focal or Diffuse	Craniectomy	Injury site	Severity	Repetitive	Methods	Wake	NREM	REM	Actigraphy
Konduru et al. (2021)	CCI	Mouse	Focal	Yes	Right temporo-parietal cortex	Moderate to severe	No	PSG	↑ Mean time at 1 month	↓ Mean time at 1 month ↑ Mean normalized delta power 1 week	↓ Mean time at 1 week	N/A
Thomasy and Opp (2019)	CCI	Mouse	Focal	Yes	Left parietal cortex	Moderate	No	PSG	↑ # Bouts during dark period ↓ % Time 15 and 30 days during dark period	↑ Delta power at 3, 7 ↑ % Time 15 and 30 days during dark period	No change % Time	N/A
Willie et al. (2012)	CCI	Mouse	Focal	Yes	Left frontoparietal	Moderate	No	PSG	↑ # Bouts during dark period ↓ Length of bouts during dark period	N/A	N/A	N/A
Hazra et al. (2014)	CCI	Mouse	Focal	Yes	Posterior to bregma right side	Mild	No	Home cage activity software	N/A	N/A	N/A	↓ Mean sleep bout length dark period vs. light ↑ Wake light boutsat week 4
Thomasy et al. (2017)	CCI	Mouse	Focal	Yes	Left parietal cortex	Mild and moderate	No	PSG	↓ Moderate TBI % time dark period day 15	↑ Moderate TBI % time NREM dark period 15 days post injury ↑ Delta power moderate 7 and 15 days light period ↑ Mild TBI delta power day 7 dark period ↑ Moderate TBI Delta power day 15 dark period ↓ Sham Delta power at day 7 dark period	↑ % timemildlight 7 and15 days↑ % timemoderatelight 7 days	N/A
Lim et al. (2013)	Fluid percussion	Mouse	Diffuse	Yes	Right parietal area	Mild	No	PSG	↓ Peak frequency	No change peak frequency	↓ Peak frequency	N/A
Skopin et al. (2015)	Fluid percussion	Rat	Diffuse	Yes	Left parietal cortex	Moderate	No	PSG	↑ # Bouts at day 6, 29 dark period ↓ Mean bout length at day 6, 29 dark period	↓ NREM total time light period at day 29 ↑ # Bouts at day 6 and 29 dark period ↓ Mean bout length at day 29 dark period	No change	N/A
Rowe et al. (2014b)	Fluid percussion	Mouse	Diffuse	Yes	Between bregma and lambda	Moderate	No	Sleep cage system	N/A	N/A	N/A	↑ % Sleep in injury and sham groups following sleep disruption
Rowe et al. (2014a)	Fluid percussion	Mouse	Diffuse	Yes	Between bregma and lambda	Moderate	No	Sleep cage system	N/A	N/A	N/A	↑ % Time sleep during the first week post-injury ↑ % Time sleep in TBI mice compared to uninjured during the dark cycle ↑ Mean length of sleep bout first week post-injury
Modarres et al. (2017)	Fluid percussion	Mouse	Diffuse	Yes	Between bregma and lambda right parietal	Mild	No	PSG	↑ Theta:alpha amplitude ratio	No change Theta:alpha amplitude ratio	N/A	N/A
Rowe et al. (2014c)	Fluid percussion	Mouse	Diffuse	Yes	Centered between bregma and lambda	Mild or moderate	No	Sleep cage system	N/A	N/A	N/A	↑ % Sleep over the first 6 h ↑ Median bout length over the first 4 h
Noain et al. (2018)	Weight drop	Rat	Mixed	No	Pre-frontocortical	Mild	No	PSG	↓ % Wake/dark period at 28 days	↑ % NREM/dark period at 28 Days ↑ Relative delta power at 7 days	No change %REM/dark period	N/A
Büchele et al. (2016)	Weight drop	Rat	Mixed	No	Anterior to bregma over the midline	N/A	No	PSG	No change % of light period	No change % of light period	No change % light period	N/A
Sabir et al. (2015)	Weight drop	Mouse	Mixed	No	Lateral right of midline posterior to bregma	Mild	No	PSG	↓ # long bouts at day 1 Relative delta activity blunted day 1 higher day 2	No change relative delta activity	Relative delta activity not reported	N/A
Vigil et al. (2023)	Blast model	Mouse	Diffuse/blast	No	Head-on	Mild	Yes (3 blasts with 24 hr intervals between each occurrence)	PSG	↓ % Time	No Change % Time	No Change % Time	N/A
Mountney et al. (2021)	Penetrating balistics model	Rat	Mixed model	Yes	Anteroposterior and medio-lateral from bregma	Severe	No	PSG	↓ Ipsilateral alpha, beta, and sigma total power vs. ipsilateral sham ↓ Ipsilateral % vigilance state light period vs. ipsilateral sham	No change total power ↑ Ipsilateral % vigilance state light period vs. ipsilateral sham	↓ Ipsilateral alpha and beta total power vs. ipsilateral sham ↓ Ipsilateral % vigilance state light period vs. ipsilateral sham	N/A
Bibineyshvili et al. (2022)	Secondary blast	Mice	Diffuse/blast	No	Right parietal region	Not reported	No	PSG	↑Delta/apha ratio high delta group 0, 1, 2, 3 days post	↑Delta/apha ratio high Delta group 0, 1, 2, 3, 4 days post	↑Delta/alpha ratio high delta group 0, 1, 2, 3 days post	N/A
Konduru et al. (2022)	CCI	Mice	Focal	Yes	Right temporo-parietal cortex	Moderate to severe	No	EEG	N/A	↑ Mean delta power 1 week and 1 month	↓ Mean time at 1 week, reverts to baseline at 1 month	N/A
Portillo et al. (2023)	Blast, Closed head injury (CHI), and rotational	Mice	Mixed models	No	Blast = frontal exposure CHI = bregma	Mild	Yes	Sleep cage system	N/A	N/A	N/A	↑ % Sleep in male injury/stress mice during the dark phase. No change for females
Korthas et al. (2022)	High frequency head impact (HF-HI) and CCI	Mice	Mixed	Yes for CCI	HF-HI: Varies CCI: center of left parietal bone	Mild (HF-HI) and Severe (CCI)	Yes for HF-HI (5 a day for 6 d)	EEG	No changes for either group	No difference in % sleep or bout length for either group	No difference in % for either group, but HF-HI led to shorter bouts during light cycle	N/A
Holden et al. (2021)	CCI	Mice	Focal	Yes	Anteroposterior and medio-lateral from bregma	Mild	No	Electrocorticography (ECoG)	N/A	Fewer spindles ipsilateral to injury site compared to contralateral side	N/A	N/A
Nichols et al. (2016)	Weight drop	Mice	Mixed	No	Centered between bregma and lambda	Mild	Yes	Sleep cage system	N/A	N/A	N/A	↑ % Total Sleep after injury
Saber et al. (2021)	Fluid percussion	Mice	Diffuse	Yes	Centered between bregma and lambda	Mild to moderate	No	Sleep cage system	N/A	N/A	N/A	↑ % Totalsleep after injury with shorter mean bout lengths
Saber et al. (2020)	Fluid percussion	Mice	Diffuse	Yes	Centered between bregma and lambda	Mild to moderate	No	Sleep cage system	N/A	N/A	N/A	↑ % Total sleep for both sexes in L/D cycles. Male TBI mice slept 11–17% more than female TBI mice
Rowe et al. (2019)	Fluid percussion	Mice	Diffuse	Yes	Centered between bregma and lambda	Mild to moderate	Yes	Sleep cage system	N/A	N/A	N/A	↑ Cumulative sleep for TBI and r-TBI mice during dark cycle and ↓ cumulative sleep during light cycle
Rowe et al. (2018)	Fluid percussion	Mice	Diffuse	Yes	Centered between bregma and lambda	Moderate	No	Sleep cage system	N/A	N/A	N/A	↑ % Sleep for vehicle TBI mice during the first light cycle
Borniger et al. (2018)	CHI	Mice	Mixed	No	Anteroposterior from bregma	Mild	Yes	No change % time	No change % time	No change % Time	No change % time	N/A
Harrison et al. (2015)	Fluid percussion	Mice	Diffuse	Yes	Centered between bregma and lambda	Mild to moderate	No	Sleep cage system	N/A	N/A	N/A	↑ Sleep bouts for TBI mice during the first light period post-injury
Petraglia et al. (2014)	Closed head acceleration-deceleration	Mice	Mixed	No	Varies	Mild	Yes	EEG	↑ % Time	↓ % NREM shift to higher frequencies ↑ bouts	No change % time	N/A
Komoltsev et al. (2021)	Fluid percussion	Rat	Diffuse	Yes	Centered on parietal bone	Mild to moderate	No	ECoG	No change	SWD occurrence higher in TBI rats 7 DPI in transition from wake to NREM	No change	No change
Andrade et al. (2022)	Fluid percussion	Rat	Diffuse	Yes	Anteroposterior and medio-lateral from bregma	Mild to moderate	No	EEG	No change % time	Average duration of N2 periods was shorter than shams during dark cycle More transitions between N3 and wake for TBI rats	↓ % Time during dark cycle	N/A

Sleep disturbances can exacerbate pathology after a TBI and lead to longer recovery times in patients, thus, treating sleep disturbances is of high priority to increase the quality of life of TBI survivors (Wiseman-Hakes et al., 2013; Theadom et al., 2016; Sandsmark et al., 2017; Morse and Garner, 2018). Unfortunately, there is a lack of effective long lasting sleep treatments for TBI (Wickwire, 2020). While the exact mechanisms responsible for sleep disturbances caused by TBI are complex and unknown, the contribution of pro-inflammatory cytokines and alteration of neurovascular hemodynamics is discussed in this review (Pop and Badaut, 2011; Viola-Saltzman and Watson, 2012; Lok et al., 2015; Sandsmark et al., 2017; Aoun et al., 2019; Green et al., 2020; Rowe and Griesbach, 2022).

## 4 Models of TBI and sleep disturbances

Sleep has been studied post-TBI in a range of animal models (Table 1). Here, we discuss sleep post-TBI after diffuse, focal, and blast injuries. Most research on sleep post-TBI focuses on the acute period post-injury and details the post-traumatic sleep response (Rowe et al., 2014c, 2019). Few studies assess sleep at chronic post-injury time points. This is an important area for investigation since individuals with TBI report sleep dysregulation occurring 6 months or longer following TBI (Baumann et al., 2007).

### 4.1 Sleep after diffuse and mixed-model TBI

It is well established that diffuse TBI causes an acute increase in sleep, known as a period of ‘post-traumatic sleep’. There is a robust increase (over 50%) in time spent asleep in the first 6 h post-injury in mice (Rowe et al., 2014c). This period of post-traumatic increase in sleep occurs regardless of injury severity or time of day the injury occurs (Rowe et al., 2014c). This increase in sleep extends to the first week after mFPI, where brain-injured mice slept more than sham controls (Saber et al., 2020). Additionally, evidence suggests that sex differences in sleep responses to TBI occur with male mice sleeping more than female mice acutely after a TBI (Saber et al., 2020). While chronic sleep disturbances post-TBI are often reported in the clinic, enhanced sleep from mFPI was not sustained chronically in the mouse (Rowe et al., 2014a). After LFPI, a mixed model of diffuse and focal TBI, acute (6 days post-TBI) and chronic (29 days post-TBI) sleep disturbances were observed (Skopin et al., 2015), although there were no differences in sleep architecture at the midpoint (19 days post-TBI).

One study found that 6 days post-LFPI mice had increased bouts of NREM sleep (Skopin et al., 2015). Differences in sleep responses to TBI in rodents are shown to occur mostly during the dark period, which might be due to ceiling effects from their increased sleep during the light period. Nevertheless, increases in EEG delta power after weight drop are observed 7 days post-injury (Noain et al., 2018). Decreased long bouts of wakefulness during the dark period 1 day after TBI and increased EEG delta activity during the second day after weight drop are reported (Sabir et al., 2015). Increased bouts of NREM sleep following LFPI have also been observed during the dark period in rats (Skopin et al., 2015).

### 4.2 Sleep after focal TBI

After CCI, mice have identifiable gliosis and changes in sleep and EEG delta power (Willie et al., 2012; Hazra et al., 2014; Thomasy et al., 2017; Thomasy and Opp, 2019; Konduru et al., 2021, 2022). Another study found that EEG delta power during NREM sleep decreased by 55% after CCI at 7 days post-injury, and theta power (5.5–8.5 Hz) increased 59% during REM sleep. However, the same study found that there were no differences in time spent awake, in REM, or in NREM sleep after CCI at 7 days post-injury (Korthas et al., 2022). At 1–2 months post-TBI, CCI mice had greater sleep efficiency and altered sleep architecture compared to sham controls (Konduru et al., 2021). CCI mice also exhibited increased epileptiform activity acutely at week 1 or chronically at 1, 2, or 3 months, which may further cause sleep disruptions (Konduru et al., 2021).

### 4.3 Sleep after blast TBI

Hypersomnia and reduced EEG gamma power during a slow-wave-sleep state post-injury was reported in one bTBI study (Vigil et al., 2023). However, the polysomnography (PSG) surgery occurred post-TBI so proper baseline normalization of EEG signals was not done and sleep was assessed 24 h after the injury where inflammation and surgical procedures could affect sleep responses (Beuckmann et al., 2019). Another bTBI and PSG study in rats reported 24 h post-injury hypersomnia effects and did not show persistent effects or determine hallmarks of bTBI sequalae (Mountney et al., 2021). A third study found hypersomnia and EEG delta/theta ratios but only assessed within 1 week post-injury (Bibineyshvili et al., 2022).

## 5 TBI-induced mechanisms that dysregulate sleep

### 5.1 Pro-inflammatory cytokines and sleep

Cytokines are small cell-signaling molecules that have multiple functions including immune, inflammatory, vasoregulatory, and sleep regulatory functions. Many pro-inflammatory cytokines can alter sleep, electroencephalogram (EEG) power spectra, and induce sleepiness, and cytokines with anti-inflammatory properties tend to attenuate sleep responses to sleep promoting stimuli (Zielinski and Gibbons, 2022). Interleukin-1 beta (IL-1β), tumor necrosis factor-alpha (TNF-α), and IL-6 are the most well characterized pro-inflammatory cytokines that regulate sleep (Kinoshita et al., 2002; Krueger, 2008; Krueger et al., 2011). These cytokines are largely enhanced in sleep regulatory brain areas, such as the cortex, brainstem, and basal forebrain, and enhance non-rapid eye movement (NREM) sleep and EEG delta power (0.5–4 Hz frequency) after stimulation by sleep loss, disease, or injury (Krueger, 2008; Krueger et al., 2011). Pro-inflammatory cytokines can also alter sleep architecture, for example, they result in suppressed or delayed rapid eye movement (REM; Krueger et al., 2011; Zielinski and Gibbons, 2022).

Inflammation can promote sleep or result in sleep fragmentation depending on the stimuli severity or dose, stimuli duration, or timing post-stimuli (Zielinski and Gibbons, 2022). IL-1β, TNF-α, and IL-6 are increased in the circulation after sleep loss (Krueger, 2008). Application of IL-1β or TNF-α directly to the brain enhances NREM sleep (Dickstein et al., 1999; Baker et al., 2005). Inhibiting these cytokines or their receptors with transgenic mice, pharmacologically, or with small interfering RNA reduces sleep responses to sleep promoting stimuli (Krueger, 2008). Moreover, nucleotide-binding domain and leucine-rich repeat protein-3 (i.e., NLRP3) inflammasomes, which are sensing protein complexes that induce the somnogenic molecules IL-1β and IL-18 and are a critical regulator or NREM sleep and EEG delta power, are activated in the brain from sleep loss, pathogens, altered metabolism, and following a TBI (Zielinski et al., 2017; Carey et al., 2023). This further implicates the role of pro-inflammatory cytokines in TBI-induced sleep dysregulation. Although speculative, it is likely that sleep dysregulation following TBI is, in part, caused by both activation and modulation of cytokines and their receptors, and the reciprocating changes in these molecules caused by altering sleep amounts and sleep fragmentation.

There is a temporal association between the acute increase in sleep observed after diffuse TBI, and levels of pro-inflammatory cytokines (Rowe et al., 2014c). Consistent with diffuse TBI models, cytokines IL-1β and TNF-α are elevated hours after injury in CCI and LFPI models of focal TBI which can potentially contribute to sleep dysregulation (Dalgard et al., 2012; Perez-Polo et al., 2013). The literature reports that bTBI animal models can increase pro-inflammatory somnogenic cytokines in the brain, such as IL-1α, IL-1β, and IL-6 that could contribute to sleep dysregulation (Heyburn et al., 2023). Additionally, patients with TBI have elevated serum levels of pro-inflammatory cytokines (Morganti-Kossman et al., 1997; Morganti-Kossmann et al., 2002), including IL-1β (Tasçı et al., 2003). These findings are consistent with studies of increased sleepiness after TBI in humans and level of TBI severity with IL-1β potentially increasing sleepiness after injury (Watson et al., 2007). Cellular and molecular mechanisms other than those involving inflammation can also contribute to the sleep effects, such as loss of hypocretin neurons post-TBI (Thomasy and Opp, 2019), changes in hypocretin ligands and receptors (Korthas et al., 2022), and changes in circadian gene expression (e.g., *Clock, Per1, Per 2, Bmal, Cry1, Cry2*; Boone et al., 2012; Govindarajulu et al., 2023).

### 5.2 The blood–brain-barrier and cerebral blood flow post-TBI

The mechanical force of TBI can cause disruption to the blood–brain-barrier (BBB) upon impact (Cash and Theus, 2020). However, secondary BBB damage can occur due to ongoing inflammatory processes including pro-inflammatory cytokine signaling (Figure 1; Cash and Theus, 2020). Both focal and diffuse brain injuries disrupt the BBB, but the type of damage differs between brain injury models (Chodobski et al., 2011). For example, CCI and LFPI cause immediate shearing of blood vessels upon impact. Diffuse and closed head injury models also cause some bleeding upon impact; however, this is not due to the penetration of a metal piston or fluid impulse, this is due to compression of the brain from acceleration/deceleration forces. Blast TBI causes widespread disruption of the BBB, consisting of many microbleeds (Kawoos et al., 2021). One study found that in 8/10 brain regions tested there was significant bleeding within 15 min of blast induced TBI in rats (Logsdon et al., 2018). Mechanistically, the diffuse BBB disruption caused by blast TBI is due to disruption of tight junctions from loss of transmembrane and adhesion molecules (Hue et al., 2013; Heyburn et al., 2019). Furthermore, increased permeability of the BBB has been observed chronically post-injury, regardless of severity (Andrews et al., 2016). Damage to cerebral vasculature can cause long term alteration in cerebral blood flow (CBF; Golding, 2002; Monson et al., 2019). Reduced CBF can worsen patient outcomes and prolong secondary inflammation post-TBI (Jenkins et al., 1989; Robertson et al., 1992). After TBI, hyperpermeability of the cerebral vasculature can cause edema which increases intracranial pressure and decreases cerebral perfusion pressure (Alluri et al., 2018).

### 5.3 Cerebral blood flow and sleep post-TBI

CBF is intrinsically related to changes in neuronal activity and functions to support the rapid need for glucose and oxygen from changing brain demands (Zheng et al., 2016). An interaction between neuronal activity and CBF is referred to as neurovascular coupling and it is tightly regulated (Iadecola, 2017). Altered CBF often accompanies TBI as early as hours after injury in the very acute post-injury phase with decreases observed in patients (Bouma et al., 1992; Soustiel et al., 2005; Salehi et al., 2017), although an increase in CBF is reported in day 1–5 post-injury and is associated with better outcome (Kelly et al., 1997). Intriguingly, CBF is also reported to be reduced at 6 and 12 months post-injury in TBI survivors compared to healthy controls (Gaggi et al., 2023). There are physiologic, neurologic, and immunologic changes that mediate changes in neurovascular hemodynamics to affect CBF and link alterations in sleep. Specifically, cytokines such as TNF-α and IL-1β can modulate CBF (Vila and Salaices, 2005). For example, chronic intracerebroventricular infusion of IL-1β reduces CBF in rodent models suggesting that chronic IL-1β might be involved in the TBI hypoperfusion (Maher et al., 2003).

### 5.4 CBF and sleep

Evidence indicates that CBF and sleep have a dynamic relationship, which alludes to the potential effects on this relationship from TBI since both CBF and sleep are altered by TBI (Viola-Saltzman and Watson, 2012; Salehi et al., 2017). Broadly, global decreases in CBF during NREM compared to wake are observed (Townsend et al., 1973; Madsen et al., 1991a,b; Braun et al., 1997; Bangash et al., 2008). A further decrease in CBF during NREM was found in humans as well as parallel findings in measurements of cerebral metabolic rate (Townsend et al., 1973; Madsen et al., 1991b; Braun et al., 1997; Bangash et al., 2008). Recent reports demonstrate both vascular diameter and hemoglobin concentrations increase in NREM and REM compared to awake brain states in mice (Turner et al., 2020). Typically, blood flow increases to areas of the brain by vessel dilation, although reductions in microvascular pressure and myogenic mechanisms can also modulate CBF (Cipolla, 2009). Consequently, it is plausible that pro-inflammatory cytokines, which can induce vasodilation, can affect hemodynamics, in part, from the modulation of cytokines from TBI (Vila and Salaices, 2005). Nevertheless, in both humans and rodents, blood flow changes during sleep states and during sleep state transitions, suggesting that altered hemodynamics from TBI could influence sleep or vice versa (Zielinski and Gibbons, 2022).

## 6 Treating TBI-induced sleep disturbances

Sleep disturbances are experienced by TBI survivors; however, treating sleep disturbances has proven challenging. Therapeutics are typically administered to mitigate the dominant symptom or treat a specific sleep–wake disorder. Clinical studies support the potential use of pharmacological compounds that promote wakefulness, such as modafinil, to treat excessive daytime sleepiness in TBI patients. Wake-promoting agents have shown some success in ameliorating excessive sleepiness after TBI (Kaiser et al., 2010; Menn et al., 2014), while stimulants have been less studied in the clinical setting. In a preclinical model of TBI, modafinil attenuates neuroinflammation and exerts neuroprotective effects (Ozturk et al., 2021). These anti-inflammatory actions may underlie the effectiveness of modafinil in treating excessive daytime sleepiness after a brain injury.

Treating the underlying pathology, as opposed to the dominant symptom, has shown efficacy in preclinical TBI models. Novel compounds that target cytokines have been administered to reduce inflammation and subsequently prevent TBI-induced sleep disturbances in the mouse (Rowe et al., 2018; Apostol et al., 2022). Importantly, when acute TBI-induced sleep is reduced with a pharmacological intervention, mice have improved functional outcomes (Rowe et al., 2018; Apostol et al., 2022). This highlights the importance of treating sleep disturbances with the goal of improving functional recovery and quality of life.

Other novel treatments for TBI-induced sleep disturbances include light therapy, dietary supplementation, and cognitive-behavioral therapy. Dietary supplementation with branched chain amino acids (BCAAs) improves wakefulness and cognition in a rodent model of TBI (Lim et al., 2013; Elkind et al., 2015). BCAA supplementation also improved insomnia severity and sleep measures determined by actigraphy, in the chronic phase of recovery from TBI in a cohort of veterans (Elliott et al., 2022). In a mechanistic study to investigate the action of BCAAs on TBI-induced sleep disturbances it was identified that BCAAs restore excitatory glutamate within presynaptic terminals on wake-promoting orexin neurons (Elliott et al., 2018).

Survivors of TBI experience medical, psychological, and mental health comorbidities during the long-term recovery process that complicate treatment strategies. Comorbidities associated with TBI include pain, headaches, and endocrine dysfunction, which further exacerbate sleep disturbances (Rowe and Griesbach, 2022). It should be noted that treatment of these comorbidities can include medications that interfere with healthy sleep. There is a critical gap in the current literature around combination therapies to treat TBI-induced sleep disturbances and comorbid symptoms. Further research is needed to elucidate effective treatment strategies for TBI survivors. A combination of therapies that include targeted pharmacological intervention, dietary supplementation, light therapy, and behavioral therapies should be explored.

## 7 Conclusion

Sleep is dysregulated post-TBI regardless of severity, and injury biomechanics which is consistent with reports in humans. There are diverse changes in sleep architecture, inflammation, and hemodynamics after TBI. Many of these differences are driven by the biomechanics of the injury model such as diffuse, focal, or blast injury, and by the brain regions injured. Understanding the complex interactions between the biological systems affected by TBI is essential in progressing both research and patient treatments.

## Author contributions

TG: Writing – original draft, Writing – review & editing. SC: Writing – original draft, Writing – review & editing. GM: Writing – original draft, Writing – review & editing. JC: Writing – original draft, Writing – review & editing. RR: Writing – original draft, Writing – review & editing. MZ: Writing – original draft, Writing – review & editing.
